# Interaction of liming and long-term fertilization increased crop yield and phosphorus use efficiency (PUE) through mediating exchangeable cations in acidic soil under wheat–maize cropping system

**DOI:** 10.1038/s41598-020-76892-8

**Published:** 2020-11-13

**Authors:** Muhammad Qaswar, Li Dongchu, Huang Jing, Han Tianfu, Waqas Ahmed, Muhammad Abbas, Zhang Lu, Du Jiangxue, Zulqarnain Haider Khan, Sami Ullah, Zhang Huimin, Wang Boren

**Affiliations:** 1 National Engineering Laboratory for Improving Quality of Arable Land, Institute of Agricultural Resources and Regional Planning, Chinese Academy of Agricultural Sciences, Beijing, 100081 People’s Republic of China; 2 Key Laboratory of Industrial Ecology and Environmental Engineering (Ministry of Education), School of Environmental Science and Technology, Dalian University of Technology, Dalian, People’s Republic of China; 3 National Observation Station of Qiyang Agri-Ecology System, Institute of Agricultural Resources and Regional Planning, Chinese Academy of Agricultural Sciences, Qiyang, 426182 Hunan People’s Republic of China; 4 Guangdong Provincial Key Laboratory for Radionuclides Pollution Control and Resources, School of Environmental Science and Engineering, Guangzhou, 510006 People’s Republic of China; 5 School of Civil Engineering, Guangzhou University, Guangzhou, 510006 People’s Republic of China; 6 Ministry of Agriculture of China, Agro-Environmental Protection Institute, Tianjin, 300191 People’s Republic of China; 7 Chinese Academy of Agricultural Sciences, Beijing, 100081 People’s Republic of China; 8 College of Agriculture, Henan University of Science and Technology, Luoyang, 471000 People’s Republic of China

**Keywords:** Environmental sciences, Agroecology

## Abstract

Low phosphorus use efficiency (PUE) is one of the main problems of acidic soil that limit the crop growth. Therefore, in the present study, we investigated the response of crop yield and PUE to the long-term application of fertilizers and quicklime (CaO) in the acidic soil under wheat–maize rotation system. Treatments included, CK (no fertilization), NP (inorganic nitrogen and P fertilization), NPK (inorganic N, P and potassium fertilization), NPKS (NPK + straw return), NPCa (NP + lime), NPKCa (NPK + lime) and NPKSCa (NPKS + lime). Results showed that, fertilizer without lime treatments, significantly (*p* ≤ 0.05) decreased soil pH and crop yield, compared to the fertilizer with lime treatments during the period of 2012–2018. Average among years, compared to the CK treatment, wheat grain yield increased by 138%, 213%, 198%, 547%, 688% and 626%, respectively and maize yield increased by 687%, 1887%, 1651%, 2605%, 5047% and 5077%, respectively, under the NP, NPK, NPKS, NPCa, NPKCa and NPKSCa treatments. Lime application significantly increased soil exchangeable base cations (Ca^2+^ and Mg^2+^) and decreased Al^3+^ cation. Compared to the NP treatment, phosphorus use efficiency (PUE) increased by 220%, 212%, 409%, 807% and 795%, respectively, under the NPK, NPKS, NPCa, NPKCa and NPKSCa treatments. Soil pH showed significant negative relationship with exchangeable Al^3+^ and soil total N. While, soil pH showed significant (*p* ≤ 0.05) positive relationship with exchangeable Ca^2+^, PUE and annual crop yield. PUE was highly negatively correlated with soil exchangeable Al^3+^. In addition, soil exchangeable Ca^2+^, pH, exchangeable Al^3+^ and available N were the most influencing factors of crop yield. Therefore, we concluded that lime application is an effective strategy to mitigate soil acidification and to increase PUE through increasing exchangeable base cations and reducing the acidic cations for high crop yield in acidic soil.

## Introduction

Inorganic fertilizers are widely used worldwide to achieve high crop yield^1,2^. Urea is the most commonly used N fertilizer, which have caused significant acidification in many parts of the world^3,4^. Application of urea fertilizer has been extended throughout the cultivated area in the China and the world due to its high N (46% N) content^5^ and low cost^6^. However, except urea other N-fertilizers such as ammonia sulfate also cause soil acidification, through generating protons during process of nitrification. Soil pH directly or indirectly influences the soil biochemical properties and influence the plant growth^7,8^.

Changes in soil acidity through fertilization can strongly influence the soil nutrient availability, plant growth and functionality of ecosystem^9,10^. The acidification of soil reflects the relative distributions of acidic cations (H^+^ and Al^3+^) and base (Ca^2+^, Mg^2+^, K^+^, and Na^+^) cations^11,12^, with the capacity to neutralize the acidic cations that mostly depend on exchangeable calcium (Ca^2+^) and magnesium (Mg^2+^) ions^13^. As the amount of H^+^ ion increases, the concentration of base cations decreases during ecosystem development^14^. Due to soil acidification, some negative effects may appear in soil such as depletion of base nutrients, high solubility of Al, Fe and Mn, which may cause toxicity in plant^15–17^.

Soil phosphorus is highly sensitive to soil pH^18^. In acidic soil, lower P use efficiency (PUE) is major problem in Chinese cropland^19,20^, which adversely affects the crop yield. In acidic soil P availability for plant uptake decreases due to P fixation with acidic cations such as Al and Fe^21^, which reduces the plant P uptake. Use of different organic and inorganic amendments has been reported in previous studies to enhance soil pH and PUE^22,23^. In a previous study, we observed that application of wheat straw or pig manure in combination with inorganic fertilizer increased the phosphatase activities and PUE^24^. Addition of manure can increase the soil pH due to alkalinity of manure^25^. However, liming is considered one of the most effective strategies to mitigate soil acidification, which can increase P availability in acidic soil^26,27^. In the several laboratory experiments, lower P solubility was observed in neutral and slightly acidic soils^28–30^. While, under field conditions, positive relationship between soil pH and P availability was observed^24^. In the field experiments that receive high P input, Al phosphate can also precipitate^31^. The theory of P adsorption on surface of oxides predicts that P solubility decreases when soil acidity increased^32,33^, and maximum adsorption (minimum solubility) of P occurs at around pH 4 for Al or Fe oxides^33^. Therefore, addition of lime to the acidic soil can reduce the oxides of Al and Fe^27^ and it can increase the P uptake for better crop production.

Over last several years, Chinese croplands have been subjected to significant acidification due to long-term inorganic fertilization. The southern subtropical area of China is dominant with arable land, playing a significant role in national grain production^34^. However acidification of soil is a major problem which limit the high crop production and nutrient use efficiency^35,36^. In addition, atmospheric deposition of N and sulfur (S) have further aggravated the problem of soil acidification in subtropical regions in southern China receiving the highest concentration^37–39^. Therefore, the main objectives of this study were to investigate relationships between soil pH, PUE and crop yield under long-term liming and fertilization in acidic soil. Quantitative assessment of the factors limiting the PUE and crop yield was performed in acidic soil under long-term wheat–maize rotation system.

## Materials and methods

### Experimental site description

**Figure 1:**
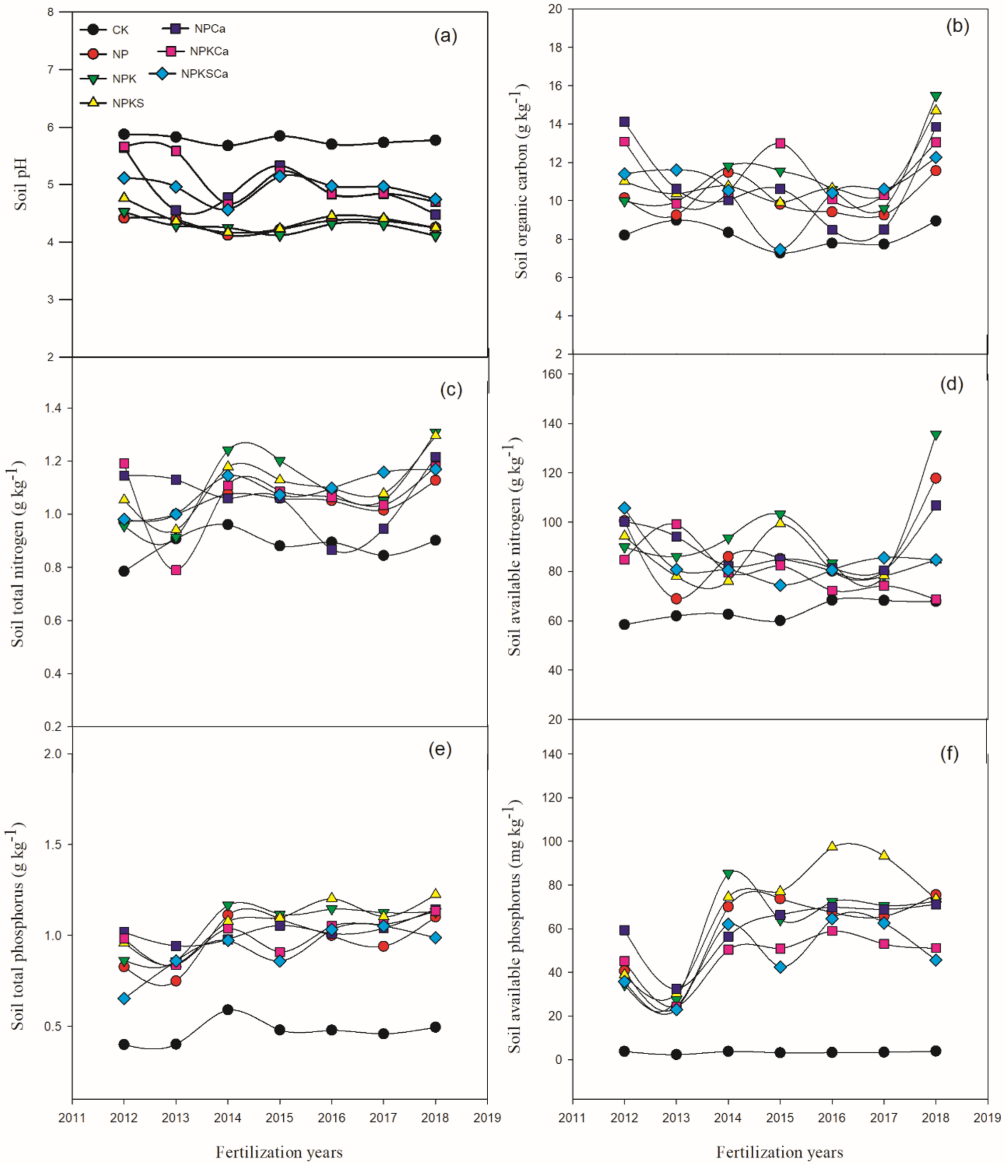
Soil pH and nutrient contents under long-term fertilization and liming in acidic soil under wheat–maize cropping system. Values are means (n = 3).

A long-term field trial was initiated in 1990 at the National observation and research station of farmland ecosystem, Qiyang county (26° 45′ 42″ N, 111° 52′ 32″ E) in southern region of China (Fig. 1). The climate at experimental site is subtropical monsoon that receives mean annual temperature of 17.8 °C and mean annual rainfall of 1290 mm. The duration of rainfall is from April to end of June every year. The climatic information during the experimental period is shown in Fig. S1, that were collected from the regional weather station following the National Standard of Specifications for Surface Meteorological Observations (1979). The soil type is Eutric Cambisol according to World Reference Base for soil resources (WRB)^40^, USDA classified this type of soil as Inceptisol with light loam soil texture and also classified as red soil based on Chinese soil classification system^41^. This soil contained 43.86% of clay content, 31.86% of silt and 24.28% of sand. The initial (1990) characteristics of topsoil (0–20 cm) included, soil pH 5.7, soil organic carbon (SOC) 7.9 g kg^−1^, total N (TN) 1.07 g kg^−1^, available N (AN) 79 mg kg^−1^, total P (TP) 0.45 g kg^−1^, available P (AP) 14.0 mg kg^−1^, total potassium (TK) 13.7 g kg^−1^ and available K (AK) was 104 mg kg^−1^.

### Experimental design and crop management

This experiment was designed under winter wheat-summer maize rotation system and the treatments were arranged in split plot design with two replicates. Each plot (20 m × 5 m) was separated from adjacent plot by 20 cm cemented baffle plates to avoid the water and treatment contamination from nearby plot. The third replication was pseudo-replication for which samples were collected from specific area in one of the original replication of each treatment according to Hurlbert^42^. The pseudo-replication in this study can increase the type 1 error in the results^43^, although there is high spatial and temporal homogeneity in the production in this field^44^. For the present study, we selected seven treatments, including (1) CK (no fertilization, control); (2) NP (inorganic N and P fertilization); (3) NPK (inorganic N, P and K fertilization); (4) NPKS (inorganic N, P and K fertilization + straw); (5) NPCa (inorganic N and P fertilization + lime); (6) NPKCa (inorganic N, P and K fertilization + lime); (7) NPKSCa (inorganic N, P, K fertilization + straw + lime). Annually, fertilizer urea was applied at the rate of 150 kg N ha^−1^, calcium superphosphate was applied at the rate of 120 kg P_2_O_5_ ha^−1^ and potassium chloride was also applied at the rate of 120 kg K_2_O ha^−1^. All fertilizers were applied before sowing, 30% and 70% of the annual inputs assigned to the wheat and maize crop, respectively. Every year, crop yield and straw were removed, while crop residues were remained in the field. In the NPKS and NPKSCa treatments, 50% of the aboveground wheat and maize straw were incorporated to the field, without considering the excess nutrients of N, P and K input through straw. In the NPCa, NPKCa and NPKSCa treatments, quick lime (CaO) was applied at the rate of 2550 kg ha^−1^ in 2010 and 1500 kg ha^−1^ in 2014 only during middle of October to mitigate soil acidification.

The experimental field was disposed of for three years before conducting experiment to ensure the same soil physical and chemical properties. Two crops were sown each year with winter wheat (Xiangmai cultivar) cultivated at the rate of 63 kg ha^−1^ (160 seeds m^−2^) followed by summer maize (Yedan-13 cultivar) at the seed rate of 60,000 seeds ha^−1^. No irrigation was applied to winter wheat and summer maize due to annual high precipitation. Pesticides Omethoate and Carbofuran were applied to control the wheat aphid during the postulation period and maize borers. Herbicide such as Glyphosate was applied to control the weeds after maize harvest. The crop was manually harvested and stubbles (about 6 cm height) and roots were remained in the soil. The collected grains and straw were air-dried and weighed separately for each crop.

### Sampling and laboratory analysis

Air-dried, grain and straw samples of crop were oven-dried at 105 °C for half hour then heated at 70 °C to a constant weight for dry matter and P content determination. Oven-dried grain and straw samples of wheat and maize crop were ground and digested with H_2_SO_4_–H_2_O_2_ at 270 °C. Phosphorus concentration in grain and straw was measured following the vanadomolybdate yellow method^45^.

Topsoil (0–20 cm) samples were collected during 2012–2018 every year after maize crop harvest from randomly selected five points in each plot using a stainless steel sampler. Composite samples were mixed thoroughly and transferred to laboratory in the clean polythene bags for chemical analysis. To measure the soil chemical characteristics, a part from composite samples was ground and sieved through 0.25-mm sieve. SOC was estimated according to oxidation method using vitriol acid potassium dichromate oxidation^46^. Concentrations of total N, P and K were analyzed in accordance with Black^47^, Murphy and Riley^48^ and Knudsen et al.^49^, respectively. Soil available N, P and K concentrations were determined according to procedures described by Lu et al.^50^ Olsen (1954) and Page et al. (1982), respectively. Exchangeable Ca^2+^ and Mg^2+^ were extracted by 1 M ammonium acetate (pH 7) and determined by atomic absorption spectroscopy. Exchangeable Al^3+^ was determined by NaOH neutralization titration after BaCl_2_ (0.1 mol L^−1^) extraction. Soil pH was determined with a glass electrode using a 2.5:1 water-soil suspension.

### Calculation

Based on amount of P fertilizer applied and P uptake by crop from 2012 to 2018, P use efficiency (PUE) in the term of P agronomic efficiency was determined for each plot using following equation^51^:

$$PUE=\frac{(YF-Y0)}{F}$$

where the PUE is phosphorus use efficiency (kg kg^−1^), YF is the annual crop yield (above-ground biomass) (kg ha^−1^) under the fertilization treatment and Y0 is annual crop yield (kg ha^−1^) under the control treatment. F is annual P input (kg ha^−1^).

### Statistical analysis

Significant differences among treatments were tested by one-way ANOVA and interaction between treatments and fertilization year were test by two-way ANOVA followed by Tukey’s HSD test at *P* = 0.05 level of significance by using statistix 8.1 (window version). Relationships between soil characteristics, PUE and crop yield were quantified by linear regression equation. Boosted Regression Tree (BRT) analysis was performed using gbm package^52^ in R version 3.3.3 to determine the relative influence of difference indexes on annual crop yield^36^. Since BRT models can incorporate both continuous and discrete explanatory variables, there is no need for prior data transformation or elimination of outliers, and they can fit complex nonlinear relationships^52^. The BRT fit was analyzed using a tenfold cross validation. BRT model was performed using tree complexity of 5 and learning rate of 0.005.

## Results

### Soil chemical properties

**Figure 2:**
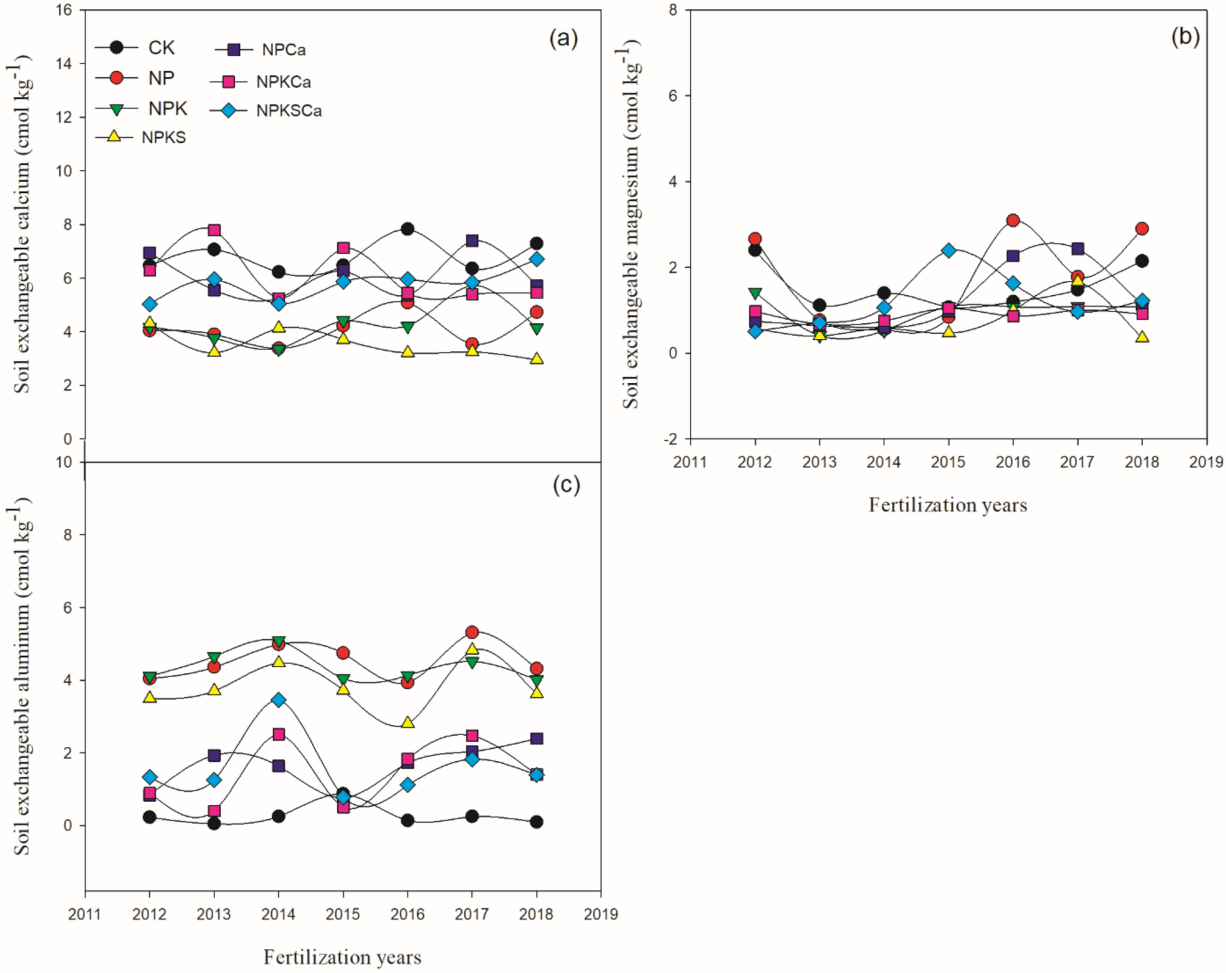
Soil exchangeable calcium (**a**), magnesium (**b**) and aluminum (**c**) cations under long-term fertilization and liming in acidic soil under wheat–maize cropping system. Values are means (n = 3).

Long-term fertilization and liming treatments significantly (*p* ≤ 0.05) affected soil chemical properties, such as pH, nutrient contents (Fig. 1) and exchangeable cations (Fig. 2). Long-term inorganic fertilization significantly decreased soil pH over the years, while, fertilizers with lime application increased the soil pH. However, soil pH was highest under the CK (control) treatment. Average across the years, soil pH under the CK treatment was 5.77. Compared to the CK treatment, soil pH under the NP, NPK, NPKS, NPCa, NPKCa and NPKSCa treatments decreased by 25.4%, 26%, 24.2%, 14.8%, 12.1% and 14.7%, respectively. Changes in SOC, total N and available N were not consistent over the years. However, in all the fertilization treatments with and without liming SOC, total N and available N contents were significantly higher than the CK treatment. On average across the years, compared to the CK treatment, the increase in SOC content was by 24.0%, 38.8%, 35.7%, 33.2%, 39.3% and 29.8%, respectively, the increase in total N was by 18.3%, 25.7%, 26.0%, 20.3%, 20.8%, and 23.6%, respectively and the increase in AN was by 38.1%, 49.7%, 32.0%,40.7%, 25.2% and 32.3%, respectively, under the NP, NPK, NPKS, NPCa, NPKCa and NPKSCa treatments. Over the years, soil total and available P content was increased in all fertilization treatments. On average, compared to the CK treatment, soil total P content increased by 107%, 130%, 128%, 118%, 113% and 95.0%, respectively, and available P increased by 1668%, 1709%, 1954%, 1699%, 1315% and 1325%, respectively, under the NP, NPK, NPKS, NPCa, NPKCa and NPKSCa treatments.

Liming with fertilization significantly increased exchangeable calcium and magnesium and decreased exchangeable aluminum compared to the fertilizer treatments without liming (Fig. 2). However, over the year, the increase in exchangeable cations were not consistent in all fertilization treatments. On average, exchangeable Ca^2+^ content was (6.8 cmol kg^−1^) highest under the CK treatment. Compared to the CK treatment, soil exchangeable Ca^2+^ content under the NP, NPK, NPKS, NPCa, NPKCa and NPKSCa treatments decreased by 39%, 37%, 48%, 11%, 10% and 15%, respectively. Averaged among years, compared to the CK treatment, exchangeable Mg^2+^ increased under the NP treatment by 16.4%, but under the NPK, NPKS, NPCa, NPKCa and NPKSCa treatments, Mg^2+^ decreased by 38%, 53%, 18.7%, 42.3% and 21.2%, respectively. Compared to the CK treatment, soil exchangeable Al^3+^ under the NP, NPK, NPKS, NPCa, NPKCa and NPKSCa treatments increased by 1576%, 1518%, 1308%, 499%, 430% and 491%, respectively.

### Crop yield, phosphorus uptake and use efficiency

**Figure 3:**
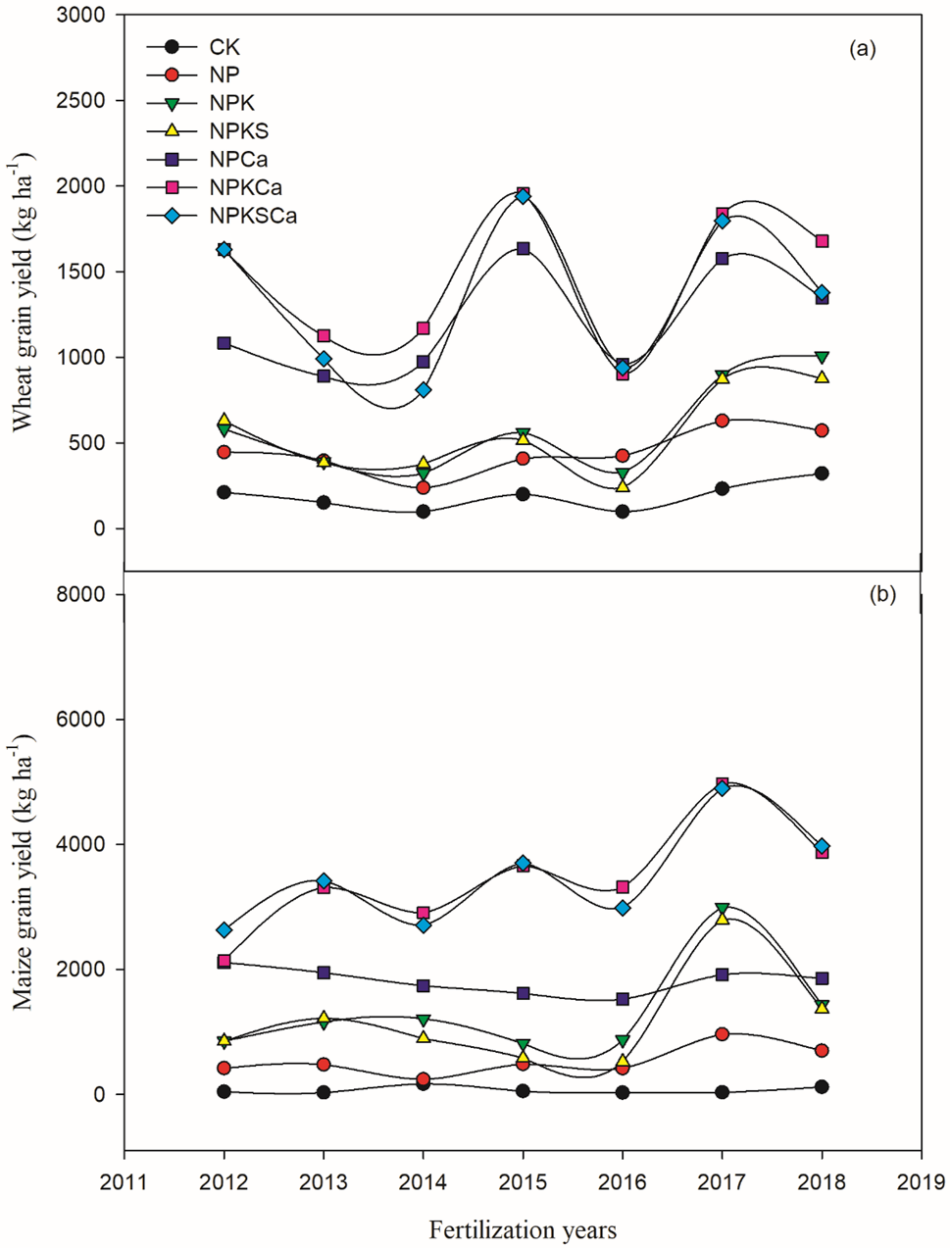
Wheat and maize yield (kg ha^−1^) under long-term fertilization and liming in acidic soil under wheat–maize cropping system. Values are means (n = 3).

**Figure 4:**
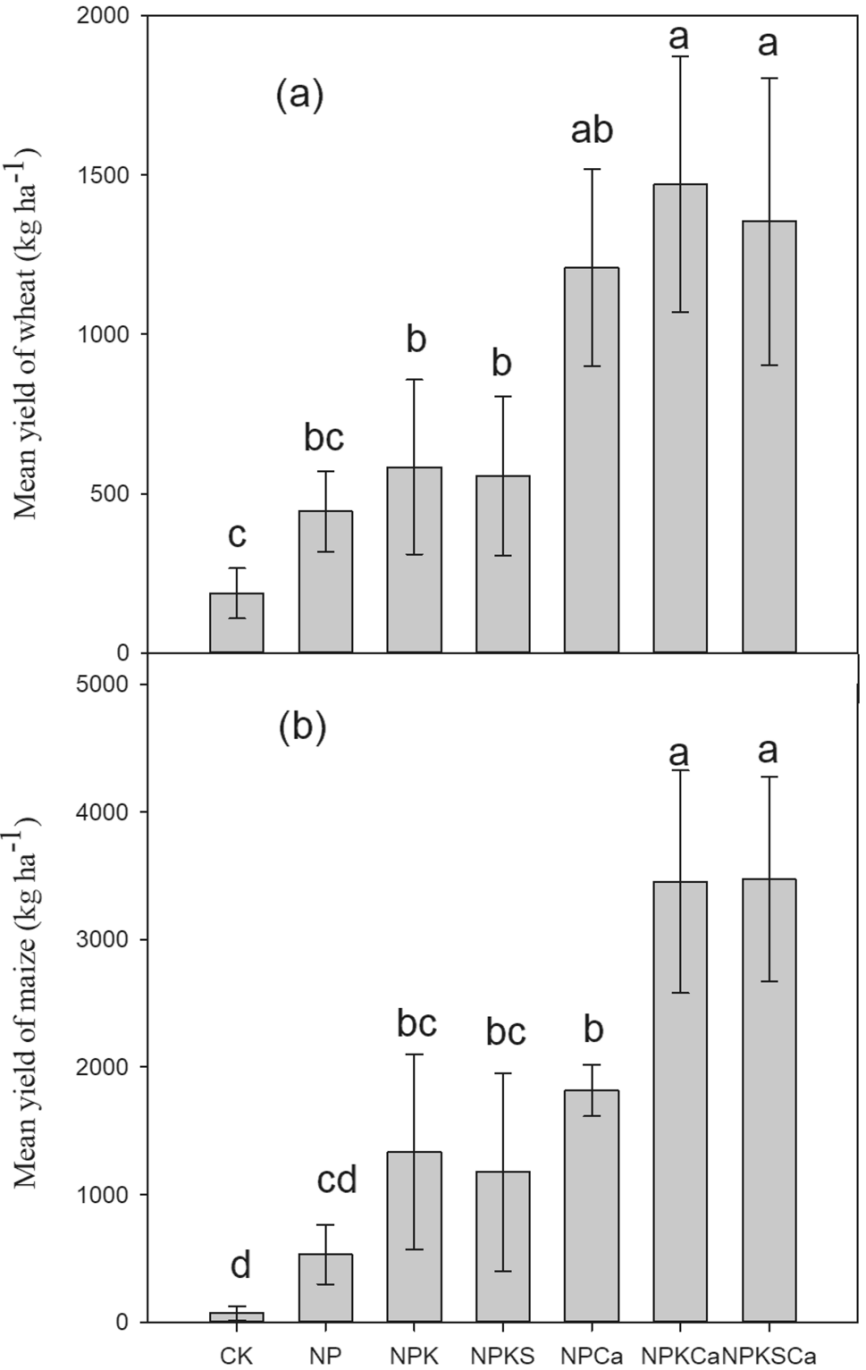
Mean grain yield of wheat (**a**) and maize (**b**) crop in each experimental plot from 2012 to 2018. Values are means of yield data from 2012 to 2018. Error bars represent the standard deviation based on data from 2012 to 2018.

**Figure 5:**
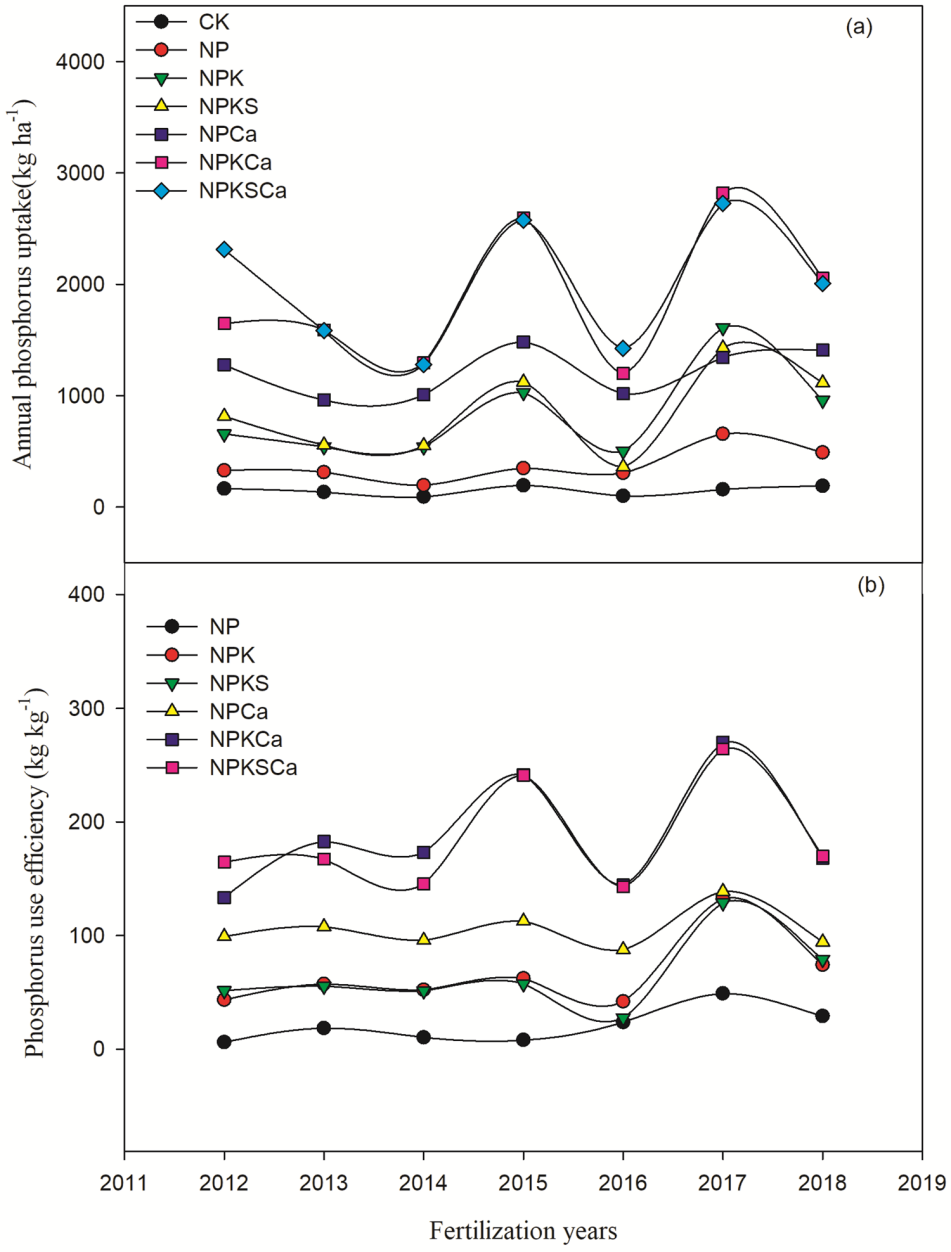
Phosphorus uptake (**a**) and phosphorus use efficiency (**b**) under long-term fertilization and liming in acidic soil under wheat–maize cropping system. Values are means (n = 3).

Long-term fertilization with lime application significantly increased wheat and maize yield compared to the fertilization without liming (Fig. 3). Both crops yield was increased over the years, especially under the NPKCa and NPKSCa treatments. On average across the years, compared to the CK treatment, wheat grain yield increased by 138%, 213%, 198%, 547%, 688% and 626%, respectively, and maize yield increased by 687%, 1887%, 1651%, 2605%, 5047% and 5077%, respectively, under the NP, NPK, NPKS, NPCa, NPKCa and NPKSCa treatments (Fig. 4). Fertilizer with lime application significantly increased P uptake and P use efficiency (PUE) during different fertilization years, compared to the fertilizer treatments without lime application (Fig. 5). Among different fertilization treatments, P uptake and PUE was highest under NPKSCa treatment. On average across the years, compared to the CK treatment, P uptake increased by 154%, 461%, 472%, 717%, 1168% and 1236%, respectively, under NP, NPK, NPKS, NPCa, NPKCa and NPKSCa treatments. On average across the years (from 2012 to 2018), PUE under the NP, NPK, NPKS, NPCa, NPKCa and NPKSCa treatments was 20.7 kg kg^−1^, 66.2 kg kg^−1^, 64.4 kg kg^−1^, 105.1 kg kg^−1^, 187.6 kg kg^−1^ and 185.0 kg kg^−1^, respectively.

### Relationships between soil pH, phosphorus use efficiency and crop yield

**Figure 6:**
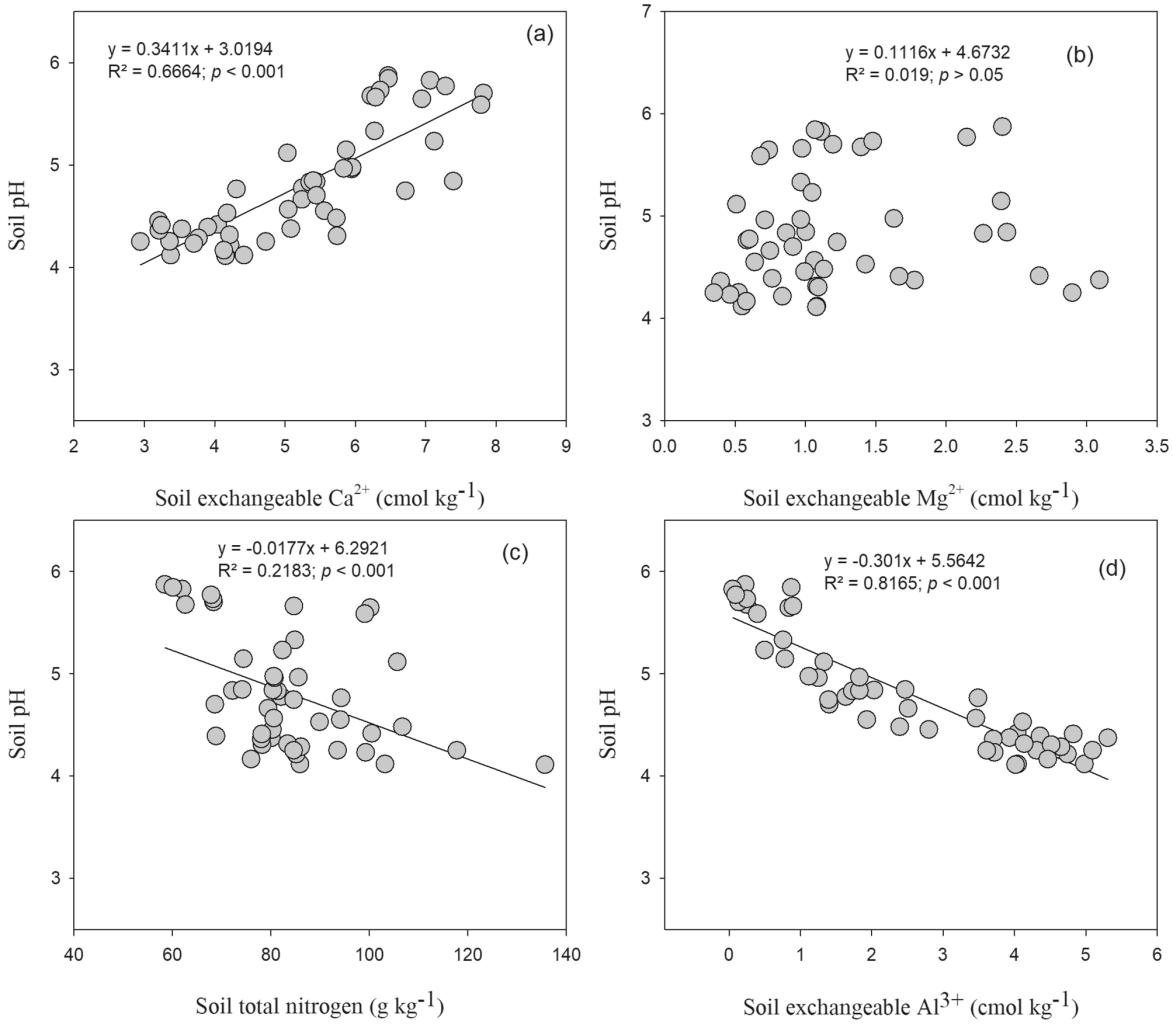
Relationship of soil exchangeable cations and total nitrogen with soil pH under long-term fertilization and liming in acidic soil under wheat–maize cropping system (n = 3).

Linear regression analysis showed that soil pH was negatively correlated with soil total N and exchangeable Al^3+^ concentrations (Fig. 6). While, significant positive relationship (*p* ≤ 0.001; R^2^ = 0.66) was observed between soil exchangeable Ca^2+^ and pH.

**Figure 7:**
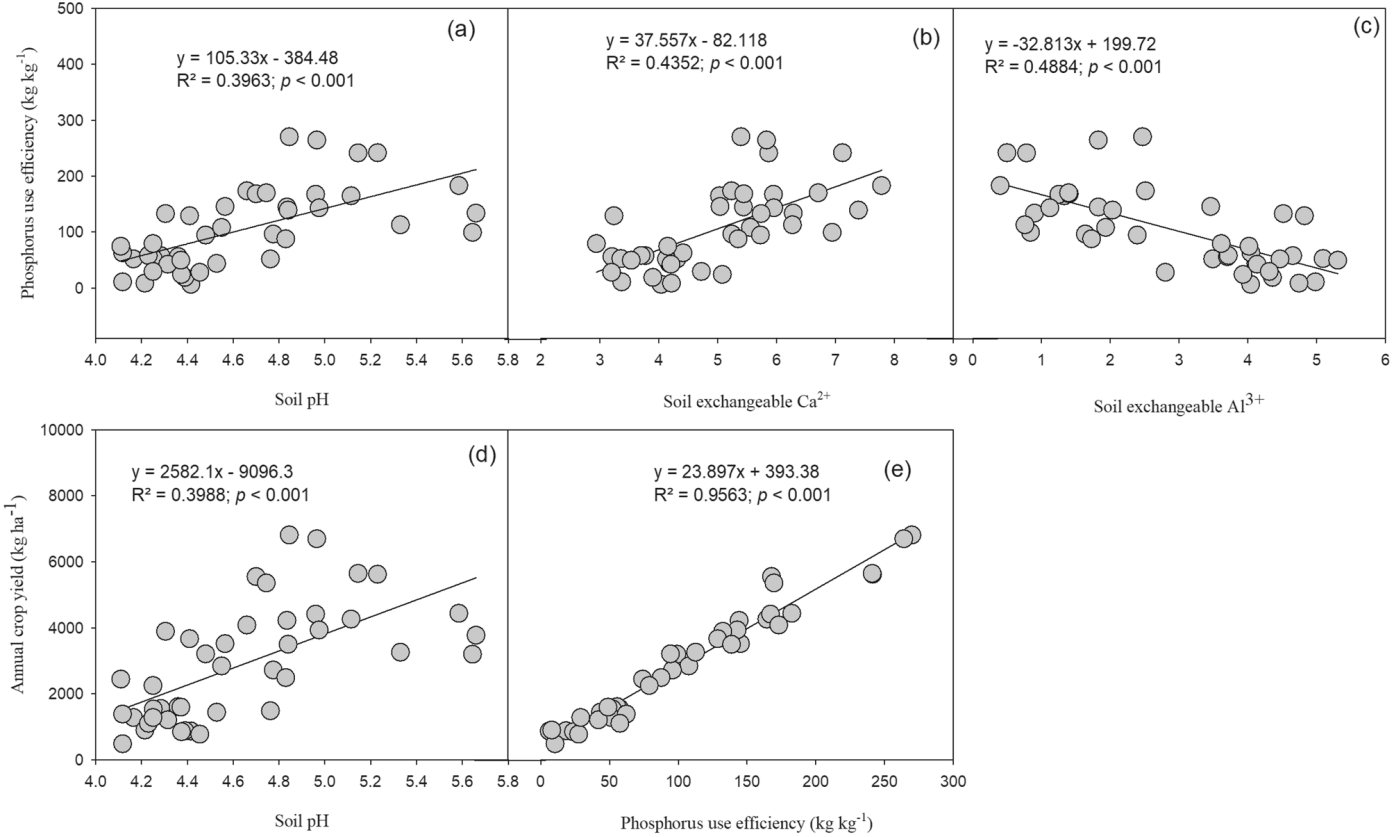
Relationships between soil pH, exchangeable cations, phosphorus use efficiency and crop yield under long-term fertilization and liming in acidic soil under wheat–maize cropping system (n = 3).

**Figure 8:**
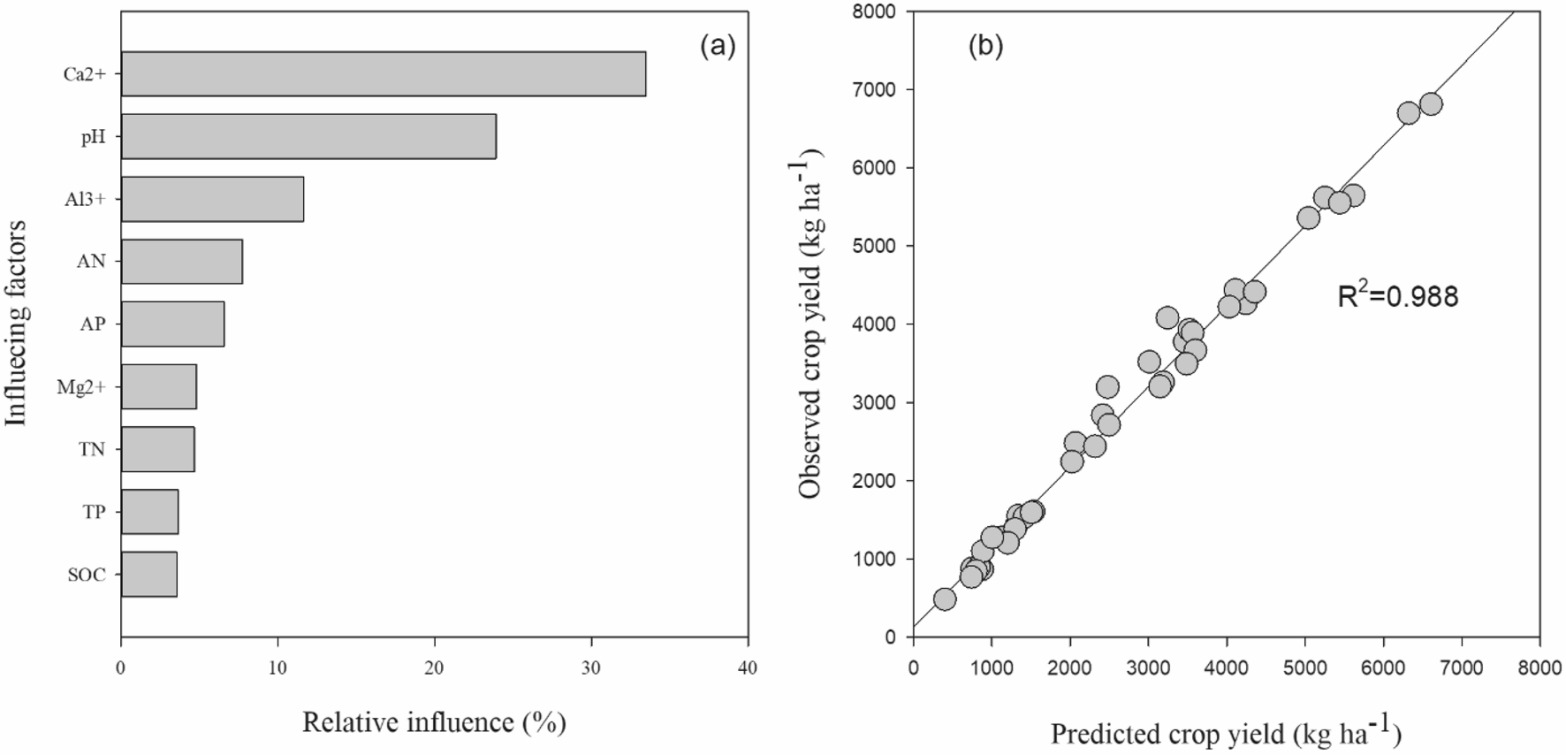
The relative contribution (%) of predictor variables for the boosted regression tree model of annual yield (**a**). Observed and predicted annual crop yield by the boosted regression tree model using predictors shown in (**b**).

Linear regression analysis showed that PUE significantly increased by increasing the soil pH and exchangeable base cation (Ca^2+^) in soil (Fig. 7). Soil pH and PUE showed significant positive relationships with annual crop yield. PUE showed significant negative relationship with exchangeable Al^3+^. Furthermore, the relative contribution of predictor variables for the boosted regression tree model of crop yield showed that exchangeable Ca^2+^, pH, exchangeable Al^3+^, available N were the most influencing factors of crop yield under the long-term liming and fertilization (Fig. 8). Relative influence of soil exchangeable Ca^2+^, pH, exchangeable Al^3+^, available N and available P on annual crop yield was 33.5%, 23.9%, 11.6%, 7.7% and 6.6%, respectively. While, relative influence of Mg^2+^, soil total N, total P and SOC was < 5%.

## Discussion

Soil acidification is one of the most important factors, limiting the high crop yield production in southern China^35,53^. In our study, long-term fertilization without lime application significantly decreased soil pH, exchangeable base cations (Ca^2+^ and Mg^2+^) and increased acidic cations (Al^3+^), while addition of lime significantly increased soil pH, base cations and decreased exchangeable Al^3+^ (Fig. 1). It has been reported in previous studies that, inorganic N fertilization induced soil acidification^54,55^, while, quicklime application reduced the soil acidification by decreasing exchangeable acidic cations effectively^27,56^. During the process of nitrification each mol of the ammonium belongs to each N-amidic and 2 mol of protons are released, which reduce the soil pH under inorganic N fertilization^57^. On the one hand, plants mostly release the net H^+^ ions; on the other hand, when anions uptake exceeds that of cations, plant release net excess of OH^−^ or HCO_3_^−^^58^. Inorganic N fertilizer application reduces the base cations in soil, which decreases the soil pH. In previous study, it was found that inorganic N fertilization shifted the soil in to the Al^3+^ buffering stage. In the acidic soil, at the soil pH below 5, hydrolysis of Al-hydroxides on the clay mineral surface release the Al^3+^ into soil solution, which decreases the base saturation cations and accelerate the soil acidification^59^. The positive effects of quicklime application on soil pH were also due to its flocculating and cementing actions^60^. Increase in soil pH might be due to precipitation of exchangeable Al and Fe as insoluble hydroxides of Al and Fe, consequently decreasing the concentrations of Al and Fe in soil solution and acidity^61^. In present study, among fertilization treatments, highest soil pH was under NPKSCa treatment that might be due to addition of lime and straw incorporation to the field. Previous studies, observed the positive effect of straw incorporation on soil pH^24^. Positive effects of straw on soil pH might be due to addition of base nutrients through straw incorporation such as Ca and K which increases the soil pH^56^.

In present study, available P in soil was higher under the NPK and NPKS treatments compared to the NPKCa and NPKSCa treatment. Soil P availability is very sensitive to soil pH^18^. In acidic soil, lower P availability could be due to P fixation with oxides of Fe and Al^62^. Application of lime may reduce the exchangeable and soluble acidic cations in soil solution and release the P in to soil solution, through changes in cation exchange capacity (CEC) and shifting phosphate adsorption–desorption equilibrium^63,64^. Therefore, in our study, soil pH showed significant negative relationship with exchangeable Al^3+^ and highly positive relationship with Ca^2+^ cation concentrations (Fig. 2). Lime application increases the microbial activities and accelerate the decomposition of organic matter which can release the inorganic P and can increase the P uptake^65,66^. In previous studies, Holland et al.^27^ observed that lime application significantly increased the soil available P in acidic soil, which was in contrast with our results (Fig. 1). Some other studies have also found that high lime application can have negative impact on soil available P due to inorganic P fixation with Ca^67^.

Acidification of soil directly or indirectly affects the soil biochemical characteristics and plant growth^7,68^. In our study, fertilization treatments with lime application significantly increased P uptake, PUE and crop yield, compared to the fertilization treatments without liming (Figs. 3, 4). These results were consistent with previous studies^27^. Kostic et al.^69^ reported that lime application to the acidic soil increased P uptake and plant available P in soil through release of root exudation of citrate in P deficient soil, which in the turn increased PUE. In another study, Shahin et al.^70^ described that effective liming of acidic soil improve plant root structure and growth, which positively influence the nutrient uptake. Poor soil fertility, nutrient losses through leaching, lower nutrient availability and accumulation of non-essential heavy metals are common characteristics of acidic soils^71,72^, which all negatively influence the plant growth and nutrients uptake. Therefore, in our study, wheat and maize crop yields under long-term fertilization without liming were very low, compared to fertilization with lime addition (Fig. 3). The highest increase in crop yield and PUE was observed under the NPKCa and NPKSCa treatment (Figs. 3, 4), that could be due to addition of lime and straw incorporation. In previous study, we found that combined application of wheat straw and inorganic fertilization significantly increased PUE by increasing P-cycling enzyme activities and P availability^24^. Increasing the soil pH through liming enhances the microbial activities^73^, which can regulate the soil P content and enhancing the P uptake. Furthermore, incorporation of crop straw improves the soil quality by increasing soil pH, improving soil organic matter (SOM) content, soil structure, aeration and retention of the high moisture content^74^, these all positive effects on soil of straw incorporation increase the crop yield. Therefore, in our study, soil pH showed significant positive relationship with PUE and crop yield (Fig. 3). Furthermore, Boosted Regression Tree (BRT) analysis showed that in acidic soil under long-term fertilization and liming, exchangeable Ca^2+^, soil pH, exchangeable Al^3+^ and available N were the most influencing factors of crop yield (Fig. 7), indicating that soil acidification highly affect the crop yield by affecting PUE. Therefore, mitigation of acidification through liming is a better approach to enhance the PUE for high crop production under long-term fertilization.

## Conclusion

We concluded that long-term fertilization without liming decreased the crop yield and PUE, because of high acidification of soil. Quicklime application significantly increased PUE and crop yield by increasing soil pH and base cations (Ca^2+^ and Mg^2+^), and reducing the exchangeable Al^3+^. Highest increase of crop yield and PUE were under the NPKCa and NPKSCa treatment, due to retention of SOC by straw and mitigation of acidification through liming. While, liming decreased soil available P in NPKCa and NPKSCa, compared to NPK and NPKS treatments, respectively. Moreover, exchangeable Ca^2+^, soil pH, exchangeable Al^3+^ and available N were the most influencing factors of annual crop yield in acidic soil. Therefore, combined fertilizer, straw and lime application could be an effective strategy to achieve high crop yield and PUE in the acidic soil under wheat–maize rotation system.

## Supplementary information

Supplementary Information.

## References

[1] Ruisi P (2016). Long-term effects of no tillage treatment on soil N availability, N uptake, and ^15^N-fertilizer recovery of durum wheat differ in relation to crop sequence. Field Crop. Res..

[2] Díaz-Zorita M, Duarte GA, Grove JH (2002). A review of no-till systems and soil management for sustainable crop production in the subhumid and semiarid Pampas of Argentina. Soil Tillage Res..

[3] Díaz-Zorita M (2005). Cambios en el uso de pesticidas y fertilizantes. Cienc. hoy.

[4] Iturri LA, Buschiazzo DE (2016). Light acidification in N-fertilized loess soils along a climosequence affected chemical and mineralogical properties in the short-term. CATENA.

[5] Finck A (1979). Fertilizer and Fertilization. Basics and Instructions for Fertilizing Crops.

[6] Smil V (2002). Nitrogen and food production: proteins for human diets. AMBIO A J. Hum. Environ..

[7] Rice KC, Herman JS (2012). Acidification of Earth: an assessment across mechanisms and scales. Appl. Geochem..

[8] Zhalnina K (2015). Soil pH determines microbial diversity and composition in the park grass experiment. Microb. Ecol..

[9] Kemmitt SJ, Wright D, Jones DL (2005). Soil acidification used as a management strategy to reduce nitrate losses from agricultural land. Soil Biol. Biochem..

[10] Zeng J (2016). Nitrogen fertilization directly affects soil bacterial diversity and indirectly affects bacterial community composition. Soil Biol. Biochem..

[11] Tian D, Niu S (2015). A global analysis of soil acidification caused by nitrogen addition. Environ. Res. Lett..

[12] Binkley D, Richter D, MacFayden A, Ford ED (1987). Nutrient cycles and H^+^ budgets of forest ecosystems. Advances in Ecological Research16.

[13] Bowman WD, Cleveland CC, Halada Ĺ, Hreško J, Baron JS (2008). Negative impact of nitrogen deposition on soil buffering capacity. Nat. Geosci..

[14] Chadwick OA, Chorover J (2001). The chemistry of pedogenic thresholds. Geoderma.

[15] Dubiková M, Cambier P, Šucha V, Čaplovic̆ová M (2002). Experimental soil acidification. Appl. Geochem..

[16] Watmough SA, Eimers MC, Dillon PJ (2007). Manganese cycling in central Ontario forests: response to soil acidification. Appl. Geochem..

[17] Neves NR (2009). Photosynthesis and oxidative stress in the restinga plant species *Eugenia uniflora* L. exposed to simulated acid rain and iron ore dust deposition: potential use in environmental risk assessment. Sci. Total Environ..

[18] Moir J, Jordan P, Moot D, Lucas R (2016). Phosphorus response and optimum pH ranges of twelve pasture legumes grown in an acid upland New Zealand soil under glasshouse conditions. J. Soil Sci. Plant Nutr..

[19] Huang Z (2016). Long-term nitrogen deposition linked to reduced water use efficiency in forests with low phosphorus availability. New Phytol..

[20] Tang X (2015). Effects of inorganic and organic amendments on the uptake of lead and trace elements by Brassica chinensis grown in an acidic red soil. Chemosphere.

[21] Redel Y (2016). Assessment of phosphorus status influenced by Al and Fe compounds in volcanic grassland soils. J. Soil Sci. Plant Nutr..

[22] Mitran T, Mani PK (2017). Effect of organic amendments on rice yield trend, phosphorus use efficiency, uptake, and apparent balance in soil under long-term rice-wheat rotation. J. Plant Nutr..

[23] Xin X (2017). Yield, phosphorus use efficiency and balance response to substituting long-term chemical fertilizer use with organic manure in a wheat-maize system. Field Crop. Res..

[24] Qaswar M (2019). Partial substitution of chemical fertilizers with organic amendments increased rice yield by changing phosphorus fractions and improving phosphatase activities in fluvo-aquic soil. J. Soils Sediments.

[25] Ahmed W (2019). Changes in phosphorus fractions associated with soil chemical properties under long-term organic and inorganic fertilization in paddy soils of southern China. PLoS ONE.

[26] Simonsson M (2018). Pools and solubility of soil phosphorus as affected by liming in long-term agricultural field experiments. Geoderma.

[27] Holland JE, White PJ, Glendining MJ, Goulding KWT, McGrath SP (2019). Yield responses of arable crops to liming—an evaluation of relationships between yields and soil pH from a long-term liming experiment. Eur. J. Agron..

[28] Weng L, Vega FA, Van Riemsdijk WH (2011). Competitive and synergistic effects in pH dependent phosphate adsorption in soils: LCD modeling. Environ. Sci. Technol..

[29] Eriksson AK, Hesterberg D, Klysubun W, Gustafsson JP (2016). Phosphorus dynamics in Swedish agricultural soils as influenced by fertilization and mineralogical properties: insights gained from batch experiments and XANES spectroscopy. Sci. Total Environ..

[30] Murrmann RP, Peech M (1969). Effect of pH on labile and soluble phosphate in soils 1. Soil Sci. Soc. Am. J..

[31] Veith JA, Sposito G (1977). Reactions of aluminosilicates, aluminum hydrous oxides, and aluminum oxide with o-phosphate: the formation of X-ray amorphous analogs of variscite and montebrasite 1. Soil Sci. Soc. Am. J..

[32] Stevens RL, Bayard E (1994). Clay mineralogy of agricultural soils (Ap horizon) in Västergötland, SW Sweden. GFF.

[33] Cabrera F, Madrid L, De Arambarri P (1977). Adsorption of phosphate by various oxides: theoretical treatment of the adsorption envelope. J. Soil Sci..

[34] Zhang H, Bo-ren W, Ming-gang XU, Ting-lu FAN (2009). Crop yield and soil responses to long-term fertilization on a red soil in Southern China. Pedosph. Int. J..

[35] Cai Z (2015). Intensified soil acidification from chemical N fertilization and prevention by manure in an 18-year field experiment in the red soil of southern China. J. Soils Sediments.

[36] Qaswar M (2020). Yield sustainability, soil organic carbon sequestration and nutrients balance under long-term combined application of manure and inorganic fertilizers in acidic paddy soil. Soil Tillage Res..

[37] Fang Y (2011). Nitrogen deposition and forest nitrogen cycling along an urban–rural transect in southern China. Glob. Change Biol..

[38] Liu L, Zhang X, Wang S, Zhang W, Lu X (2016). Bulk sulfur (S) deposition in China. Atmos. Environ..

[39] Jia Y (2014). Spatial and decadal variations in inorganic nitrogen wet deposition in China induced by human activity. Sci. Rep..

[40] FAO. *World Reference Base for Soil Resources 2014: International soil classification systems for naming soils and creating legends for soil maps (Updated 2015)*. *World Soil Resources Reports No. 106* (2014).

[41] Baxter S (2007). World reference base for soil resources. Exp. Agric..

[42] Hurlbert SH (1984). Pseudoreplication and the design of ecological field experiments. Ecol. Monogr..

[43] Guo Y (2017). Long-term grazing affects relationships between nitrogen form uptake and biomass of alpine meadow plants. Plant Ecol..

[44] Zhou H (2006). Stability of alpine meadow ecosystem on the Qinghai-Tibetan Plateau. Chin. Sci. Bull..

[45] Nelson DW, Sommers L (1982). Total carbon, organic carbon, and organic matter. Methods Soil Anal. Part 2 Chem. Microbiol. Prop..

[46] Pages AL, Miller RH, Dennis RK (1982). Methods of Soil Analysis. Part 2 Chemical Methods.

[47] Black CA (1965). Methods of Soil Analysis Part II. Chemical and Microbiological Properties.

[48] Murphy J, Riley JP (1964). A modified single solution method for the determination of phosphate in natural waters. Anal. Chim. Acta.

[49] Knudsen D, Peterson GA, Pratt PF, Norman AG (1982). Lithium, sodium, and potassium. Methods of Soil Analysis. Part 2. Chemical and microbiological properties.

[50] Lu RK (2000). Analytical Methods of Soil Agricultural Chemistry.

[51] Pavinato PS, Rodrigues M, Soltangheisi A, Sartor LR, Withers PJA (2017). Effects of cover crops and phosphorus sources on maize yield, phosphorus uptake, and phosphorus use efficiency. Agron. J..

[52] Elith J, Leathwick JR, Hastie T (2008). A working guide to boosted regression trees. J. Anim. Ecol..

[53] Guo JH (2010). Significant acidification in major Chinese croplands. Science.

[54] Schroder JL (2011). Soil acidification from long-term use of nitrogen fertilizers on winter wheat. Soil Sci. Soc. Am. J..

[55] Chen D, Lan Z, Hu S, Bai Y (2015). Effects of nitrogen enrichment on belowground communities in grassland: relative role of soil nitrogen availability vs. soil acidification. Soil Biol. Biochem..

[56] Han T (2018). The links between potassium availability and soil exchangeable calcium, magnesium, and aluminum are mediated by lime in acidic soil. J. Soils Sediments.

[57] Bouwman AF, Van Vuuren DP, Derwent RG, Posch M (2002). A global analysis of acidification and eutrophication of terrestrial ecosystems. Water. Air Soil Pollut..

[58] Tang C (2011). Biological amelioration of subsoil acidity through managing nitrate uptake by wheat crops. Plant Soil.

[59] Stevens CJ, Dise NB, Gowing DJ (2009). Regional trends in soil acidification and exchangeable metal concentrations in relation to acid deposition rates. Environ. Pollut..

[60] Haynes RJ (1982). Effects of liming on phosphate availability in acid soils. Plant Soil.

[61] Nierop KGJJ, Jansen B, Verstraten JM (2002). Dissolved organic matter, aluminium and iron interactions: precipitation induced by metal/carbon ratio, pH and competition. Sci. Total Environ..

[62] Li H (2015). Past, present, and future use of phosphorus in Chinese agriculture and its influence on phosphorus losses. Ambio.

[63] Smyth TJ, Sanchez PA (1980). Effects of lime, silicate, and phosphorus applications to an oxisol on phosphorus sorption and ion retention. Soil Sci. Soc. Am. J..

[64] Curtin D, Syers JK (2001). Lime-induced changes in indices of soil phosphate availability. Soil Sci. Soc. Am. J..

[65] Barth VP (2018). Stratification of soil chemical and microbial properties under no-till after liming. Appl. Soil Ecol..

[66] Abdi D (2016). Residual effects of paper mill biosolids and liming materials on soil microbial biomass and community structure. Can. J. Soil Sci..

[67] Park J-S, Ro H-M (2018). Early-stage changes in chemical phosphorus speciation induced by liming deforested soils. J. Soil Sci. Plant Nutr..

[68] Malhi SS, Nyborg M, Harapiak JT (1998). Effects of long-term N fertilizer-induced acidification and liming on micronutrients in soil and in bromegrass hay. Soil Tillage Res..

[69] Kostic L (2015). Liming of anthropogenically acidified soil promotes phosphorus acquisition in the rhizosphere of wheat. Biol. Fertil. Soils.

[70] Shahin, M., Esitken, A. & Pirlak, L. The effects of lime does on some morphological and fruit characteristics of some strawberry (Fragaria Xananssa Duch.) cultivars. In *IX International Scientific Agriculture Symposium" AGROSYM 2018", Jahorina, Bosnia and Herzegovina, 4–7 October 2018. Book of Proceedings* 575–582 (University of East Sarajevo, Faculty of Agriculture, 2018).

[71] Rheinheimer DS, Tiecher T, Gonzatto R, Zafar M, Brunetto G (2018). Residual effect of surface-applied lime on soil acidity properties in a long-term experiment under no-till in a Southern Brazilian sandy Ultisol. Geoderma.

[72] Goulding KWT (2016). Soil acidification and the importance of liming agricultural soils with particular reference to the United Kingdom. Soil Use Manag..

[73] Liu XY, Rashti MR, Esfandbod M, Powell B, Chen CR (2018). Liming improves soil microbial growth, but trash blanket placement increases labile carbon and nitrogen availability in a sugarcane soil of subtropical Australia. Soil Res..

[74] Yadvinder-Singh B-S, Timsina J (2005). Crop residue management for nutrient cycling and improving soil productivity in rice-based cropping systems in the tropics. Adv. Agron..

