# Supplementary material for: Interaction of liming and long-term fertilization increased crop yield and phosphorus use efficiency (PUE) through mediating exchangeable cations in acidic soil under wheat–maize cropping system
Source: Sci Rep. 2020 Nov 13;10:19828. doi: 10.1038/s41598-020-76892-8 (PMC7666156; doi:10.1038/s41598-020-76892-8)
Supplement: Supplementary file 1 — Supplementary Information. [file 41598_2020_76892_MOESM1_ESM.docx]

**Interaction of liming and long-term fertilization increased crop yield and phosphorus use efficiency (PUE) through mediating exchangeable cations in acidic soil under wheat-maize cropping system**

Muhammad Qaswar^a,b^, Li Dongchu^a,c^, Huang Jing^a,c^, Han Tianfu^a^, Waqas Ahmed^d,e^, Muhammad Abbas^a^, Zhang Lu^a^, Du Jiangxue^a^, Zulqarnain Haider Khan^f^, Sami Ullah^a^, Zhang Huimin^a,g^, Wang Boren^a,c^

^a^National Engineering Laboratory for Improving Quality of Arable Land; Institute of Agricultural Resources and Regional Planning, Chinese Academy of Agricultural Sciences, Beijing 100081, China

^b^Key Laboratory of Industrial Ecology and Environmental Engineering (Ministry of Education), School of Environmental Science and Technology, Dalian University of Technology, Dalian, China

^c^National Observation Station of Qiyang Agri-ecology System; Institute of Agricultural Resources and Regional Planning, Chinese Academy of Agricultural Sciences, Qiyang, Hunan 426182, China

^d^Guangdong Provincial Key Laboratory for Radionuclides Pollution Control and Resources, School of Environmental Science and Engineering, Guangzhou-510006, China

^e^School of Civil Engineering, Guangzhou University, Guangzhou 510006, PR China

^f^Agro-Environmental Protection Institute, Ministry of Agriculture of China, Tianjin, 300191, Chinese Academy of Agricultural Sciences, Beijing 100081, China

^g^College of Agriculture, Henan University of Science and Technology, Luoyang 471000, China

*For correspondence: [zhanghuimin@caas.cn](mailto:zhanghuimin@caas.cn), [wangboren@caas.cn](mailto:wangboren@caas.cn)

Muhammad Qaswar and Li Dongchu equally contributed to this article

Fig. S1, Mean annual temperature (MAT) and mean annual precipitation (MAP) from 2008 to 2018 at study area.
